# Engaging a rural community in identifying determinants of low birth weight and deciding on measures to improve low birth weight: an experience from a Sri Lankan study

**DOI:** 10.1186/s41043-017-0118-9

**Published:** 2017-12-06

**Authors:** N. D. Galmangoda Guruge, M. Goonasekara, S. D. Dharmaratne, M. W. Gunathunga

**Affiliations:** 1 Department of Health Promotion, Faculty of Applied Sciences, Rajarata University of Sri Lanka, Mihintale, Sri Lanka; 2 Department of Biological Sciences, Faculty of Applied Sciences, Rajarata University of Sri Lanka, Mihinthale, Sri Lanka; 3 Department of Community Medicine, Faculty of Medicine, University of Peradeniya, Peradeniya, Sri Lanka; 4 Department of Community Medicine, Faculty of Medicine, University of Colombo, Colombo, Sri Lanka

**Keywords:** Low birth weight, Community engagement, Determinants, Rural, Sri Lanka

## Abstract

**Background:**

Involving communities in identifying and addressing determinants of their own health is effective in addressing complex problems, such as low birth weight (LBW). LBW is an important public health problem which has not improved significantly in Sri Lanka in the last 10 years. This study reports the ability of lay persons to identify and address determinants of LBW.

**Methods:**

A health promotion intervention was conducted among 403 mothers registering at 26 antenatal clinics in the district of Anuradhapura, in Sri Lanka. The components of a health promotion process—initiation, maintenance and continual monitoring, and re-direction towards greater effectiveness—were explained to the mothers. Inputs were initially provided through different methods to enable mothers’ groups to identify determinants of LBW and to decide actions to address those identified determinants. The overall study was carried out over a period of 1 year, of which the intervention phase took around 7 months. The mothers in the clinic group were encouraged to continue an ongoing process in smaller “neighborhood action committees” (NACs)—of which there were 71. The findings are based on field notes maintained during the process, analyzed using thematic analysis.

**Results:**

Each group of mothers identified at least eight determinants of LBW at the first attempt (without first author’s guidance), four of which corresponded with those already mentioned in published studies. Up to five other determinants were agreed, after facilitation by the first author, at the second attempt. Of the total, 10 determinants of LBW were finally prioritized. Twenty actions to address the 10 selected prioritized determinants were agreed through a collective consensus development process.

**Conclusions:**

Lay communities successfully identified determinants of LBW and household level actions to address these, with relatively simple guidance, when stimulated to initiate the relevant process. This capacity should be nurtured and better used in interventions to improve LBW.

## Background

Birth weight is an important determinant of an individual’s health and a globally accepted predictor of childhood survival [1, 2], and improving birth weight is a critical step in breaking the life cycle effect of under-nutrition [3].

Prevalence of low birth weight (LBW) is the proportion of live births with a weight less than 2500 g, as a percentage of the total live births during a defined period. Current global prevalence of low birth weight is 15.5% and is considered a complex public health problem [3]. Even though Sri Lanka has good health indicators, LBW has remained high at a prevalence of 16–18% [4].

LBW has a wide range of determinants operating at individual, household, community, and society levels, in different stages of the life cycle. There are many potential modifiable factors related to dietary intake and care practices of the pregnant mother that can be effectively addressed during the antenatal period to prevent LBW [5–10].

As most of those determinants are dependent on the family as well as the wider community, service-provider-oriented interventions alone will not be adequate to address the problems of low birth weight during pregnancy period. Thus, simple but effective complementary public health interventions should be searched for and developed, to address the complex, interrelated household and community level determinants of LBW in the antenatal period. Global evidence suggests that programs using an “empowerment model” of health promotion, which involves communities in identifying and addressing determinants, are effective in addressing complex problems and making sustainable changes [11]. Thus, there was a need to explore ways to create such behavioral changes to address determinants of LBW during pregnancy, which are easy to integrate and implement as complementary to the current interventions of the Maternal and Child Health Programme of the Ministry of Health.

Identifying such behaviors and practices to address determinants influencing different aspects of their own health and wellbeing by lay people are crucial aspects in the effort to improve it. The ability of lay people to identify such behaviors and practices and implement those to address identified determinants of their health needs to be tested.

Health promotion is a cost-effective approach for improving wellbeing of people which was recognized by the World Health Organization. This reiterates the need and importance of identifying and deciding on actions to address determinants influencing one’s own health by the particular individual or the community [11]. The health promotion approach utilized during this study in Sri Lanka involves lay people in each step of the health promotion process including identifying and addressing determinants of a particular issue or a problem [12–16]. No published data is available where the community or the lay people accomplishing these more involved aspects of promotion of their own health in Sri Lanka. This paper reports the ability of lay persons to identify and address determinants of low birth weight through community empowerment through health promotion process model. It is part of a larger study undertaken to assess the effectiveness of a community-based health promotion program on improving birth weight in the District of Anuradhapura.

## Methods

A community-based participatory approach was used.

### Setting and participants

Anuradhapura district in North central province was selected purposively to pilot the approach, considering the feasibility of implementation. It is predominantly a rural community where the main occupation of males is farming while most of the women are housewives. The study population was pregnant mothers attending field antenatal clinics or combined clinics (clinics that deliver antenatal and child welfare services together) of the Ministry of Health in the Anuradhapura district. Two classes of women come to the clinic—pregnant mothers and those who come to show their children under 5 years of age. Only pregnant mothers were subject to study but other mothers too joined the sessions conducted for the pregnant mothers. They (mothers who came to the clinic with their children) simply participated in the discussion because they were also present in the clinic at the time and all those who were interested were included in the discussion.

### Sampling

A systematic sampling method was used to recruit participants from Anuradhapura district. The primary sampling unit was Medical Officer of Health (MOH) area and the secondary sampling unit was antenatal clinic (ANC).

Three MOH areas were selected randomly using the population maps available at the Department of Census and Statistics.

The total sample size (*N* = 403) was proportionately drawn from the three MOH areas according to the population proportion of pregnant women registered in each area in the preceding quarter.

Then, the number of pregnant women allocated into each MOH area was divided by the number of pregnant women registered in the smallest ANC in the preceding quarter to decide the number of ANCs to be selected from each MOH area.

Then, the ANCs were selected randomly by lottery method using the ANC lists of each MOH area as sampling frames.

### Inclusion and exclusion criteria

All pregnant women registered in the 3rd and 4th quarters of the year 2012 in the selected ANCs were included in the study. Pregnant women registering in the ANC after 12 weeks of period of amenorrhea (POA) and pregnant women with diagnosed medical conditions/co-morbidities at the time of registration (because they get special care from the routine system) were excluded from the study.

### Data collection

Two trained research assistants with a university degree in health promotion were recruited for data collection. They were trained to observe and record both verbal and non-verbal communications during interactive sessions between the first author and mothers groups. One research assistant took all the notes from the beginning to end of each session while the other subjectively measured and kept records about participants’ apparent interests, enthusiasm, and participation.

### Intervention with mothers

The process was initiated by the first author in the clinics. The interaction with pregnant women, for this study, was conducted mostly after the routine clinic work was completed by the regular clinicians. The PHM (Public Health Midwife) too supported this activity by previously informing the pregnant mothers about the session on LBW and by attending early to the pregnant mothers who were to participate for this study. All pregnant mothers were able to finish their routine ANC activities early, as a result.

Inputs were provided using a range of different methods to initiate, maintain the process, and direct it towards achieving objectives. Twenty-six initial training sessions were conducted in 26 antenatal clinics. An average of 3 h was taken for each session. Mothers were provided with a snack and refreshments. The number of pregnant mothers varied from around 10 to 20 in each clinic.

Other mothers who came to the clinic (with their children under 5 years) also joined the discussion out of interest. They were invited to participate although not part of the study population. The number of pregnant mothers who left the discussion before the end was negligible—although they were free to leave at any time. This was probably because they realized they were getting useful knowledge and hence engaged with interest. They participated in discussions very enthusiastically and were generally quite keen not to miss any of the ideas discussed.

Study was done for 1 year, of which the intervention with mothers’ groups took an average of around 7 months.

### Conceptual frame work for the process of intervention

**Fig. 1:**
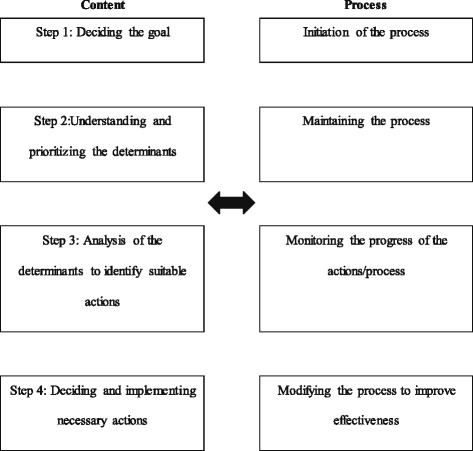
Health promotion intervention model utilized to develop and implement the intervention to improve birth weight

The conceptual framework for the process of the intervention was based on the community-centered health promotion intervention model described by Samarasinghe and colleagues in 2011 (Fig. 1).

**Fig. 2:**
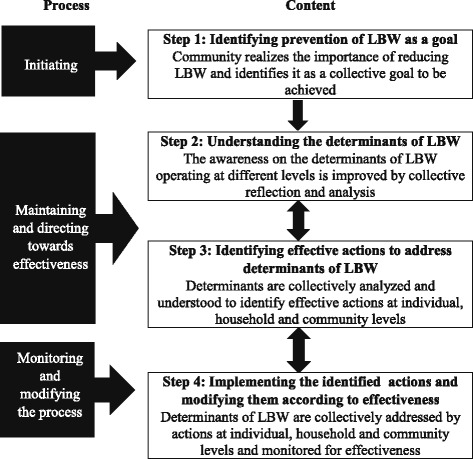
Conceptual framework for the process of community centered health promotion intervention model [12]

The adopted model (Fig. 2) consisted of integrating the components of content and process of community-centered health promotion intervention model. “Content” illustrates the core subject matter while the “process” illustrates the flow of developments through the health promoter’s interaction with the group. Even though integrated, they would be described separately for the purpose of clarity.

The model assumes that in step 1 the community or group will be able to realize the importance of reducing LBW and identify it as a collective goal to be achieved, leading to the step of identifying determinants. Awareness of the determinants of LBW operating at different levels was improved by collective reflection and analysis in step 2. Deciding actions to address identified determinants is dealt with in step 3.

The process of identifying determinants was started with a lecture discussion which aimed at identifying “improving birth weight” as a collective goal (step 1; Fig. 1). The topics discussed during advocacy on the importance of normal weight at birth are shown in Table 1.

**Table 1:** Advocacy on the importance of having normal weight at birth

1. The mother’s vision or “dream” about the child to be born 2. Importance of the first 1000 days in an individual’s life in determining his/her potential and health 3. How the first 280 days (the antenatal period) influence a child’s life 4. How birth weight is an important component of the first 1000 days of life 5. Ability to improve “birth weight” by modifying determinants operating at individual, household and community levels	

The lecture discussions were initiated with discussions about the mother’s vision or dream about the child to be born. The facilitator broadened the understanding or the vision of the pregnant mothers’ on their future expectations and hopes for the baby to be born. The kind of person they want their child to grow up to be was discussed with mothers’ groups. Mothers’ ideas included that they want the child to be intelligent, healthy, and able to succeed in life. In discussion with the first author, the vision was broadened to include having good physical, mental, and social wellbeing and not only getting a good job. The relevance of good birth weight to achieving this goal was also discussed.

That the future vision for the baby to be born should not only be of the mother but also of the father was discussed. With the broadened vision on bringing up a child with better physical, mental, and social wellbeing rather than having a child only becomes person doing a white collar job, mothers became enthusiastic about the initiated process and keen to engage with future activities of the process.

The importance of the first 1000 days in an individual’s life in determining his/her potential and health was further explained as the path to achieve the parents’ dream for their child. How the first 280 days or the antenatal period influences a child’s life was extensively discussed in order to make mothers understand the importance of the antenatal period in determining her child’s life. This led mothers to realize how birth weight is an important parameter in the first 1000 days of life which determines future of the child. After realizing normal birth weight as an important parameter to be achieved, mothers became more enthusiastic to see ways of achieving it.

When mothers realized the importance of good intra uterine growth, they became interested in learning how to ensure it. This enthusiasm was then directed towards how good growth and birth weight could practically be achieved. When the need of improving birth weight was appreciated and the possibility of doing so was seen as achievable, they were interested in understanding the way to achieve it. At this stage, discussion was directed to examining underlying factors as a step in improving birth weight. The first author then explained how determinants operate at individual, household, and community levels. This was the first session conducted during this process; it was used also to engage with the participants.

In the step 2 of the frame work, the first author led the discussions towards making the participants aware on the determinants of LBW operating at different levels, through improved collective reflection and analysis by the participants. Even though both sessions were predominantly participants’ collective reflection and analysis, there were inputs from the first author on determinants already identified for low birth weight.

Session 1 continued further with the motivation and the enthusiasm of mothers to understand determinants. The first author initially asked participants to speak up about all the determinants that came to their mind and to list these—to make it easy to revise and add new determinants, without active intervention of the first author. The research assistants kept records of each list that emerged from the discussions. This activity ended with a listing of determinants, many of which were rather superficial.

During the second half, the first author facilitated the discussion to explore more underlying factors through an assignment to seek deeper underlying determinants, beyond the superficial determinants they came up with, in the initial stage. Session 2 was initiated by revising determinants identified at the first session. The discussions were then directed to improve participants’ analysis of “determinants”. They were asked how they could determine more superficial and deeper underlying determinants, or less obvious determinants, by asking specific probing questions.

Mothers were asked to discuss the deeper underlying determinants in groups. Each group came up with a list of determinants, which they were asked to prioritize within the group. The first author listed all of these on a white board, according to each group’s perceived importance, after which they were asked to select the five most important determinants.

Then, the first author presented the participants a list prepared by him, giving determinants for LBW mentioned in the scientific literature and asked them to discuss these too, within their groups. This session was facilitated by using video clips and case scenarios, where applicable. At the end of the session, the participants agreed upon up to four determinants as priority concerns they should address, in order to reduce LBW in their community. The first author then asked the participants to formulate a final list of the 10 most important determinants.

### Deciding actions to address identified determinants

As a result of steps 1 and 2 of the model, 10 determinants for low birth weight in their own communities were identified by mothers’ groups through the facilitation of the first author.

Session 2 was then continued, to identify actions to address determinants of LBW (step 3; Fig. 1). The participants were given the opportunity to divide into smaller groups according to their area of residence or any other way they would find it convenient to work together after this session. They formed such “neighborhood action committees” (NAC) to decide on which determinants to be addressed by their committee, and by which actions.

Mothers addressed in the larger clinic groups thereby split themselves into these smaller NACs to enable them to meet regularly and discuss progress. The first author or the trained research assistants visited the local village to participate in many of these discussions. There would have been around five pregnant mothers each, in these NACs. They were joined by their husbands and family members and others from the community in these local meetings. Thus, follow-up action from the meetings with the 26 mothers’ groups formed at the clinic level happened in these local committees, which kept meeting frequently in their own locality. The number of these smaller NACs was 71.

The first author technically corrected the actions they decided to address determinants. Plans were made within the committees on implementing the selected actions, initiating step 3 of the intervention. The first author provided technical inputs, with special attention to promote collective actions as opposed to individual actions. He also ensured that selected actions included at least one measure of their own progress. Role plays and demonstrations on how to implement actions were used to improve the participants’ confidence in initiating the actions and strengthen their capacities. In subsequent sessions, success stories from other local community settings which addressed the identified determinants were presented as examples to stimulate collective reflection.

At the end of the session, the neighborhood action committees were equipped with action plans and monitoring mechanisms and a planned date for the next meeting—among themselves. Further, the progress in husband’s participation was also monitored. The pregnant mothers in addition presented their progress of family bonding during each following session.

A contact person was identified from the committee to liaise with the first author and network with the other committees for further inputs and actions.

Further follow-up sessions were conducted with each of the larger 26 mothers’ groups in the clinics, in the latter part of this process. A similar methodology was followed in the facilitation at each clinic. The meetings took place on average after 3 months. Husbands and family members were invited for at least one follow-up discussion at the clinic as well, in addition to their participation in the local NAC discussions. Participation of husbands was encouraged through the fact that mothers were engaged in monitoring the progress of their husbands in providing them with care. The husbands may have responded positively also because there was a community-wide change, due to other families in the locality also taking part in the local meetings. All husbands in the community would have been aware that the pregnant mothers were monitoring progress of family bonding.

Data collection was conducted throughout the process from the field notes of research assistants. After each session, relevant data and notes were collected from research assistants. The field notes were analyzed by two independent analysts through thematic analysis method and emerged themes were compared and restructured (if required) with the consensus of both. This paper describes its findings based on collected data.

## Results

### Participants

Pregnant mothers were selected from three MOH areas to identify determinants and actions to address low birth weight (Table 2).

**Table 2:** Participants from each MOH area

MOH area	No of participants
NuwaragamPalatha East	196
Madawachchiya	127
Mihintale	80

### Process indicators

Mothers were graded by the research assistants according to the apparent enthusiasm towards the process. There were three pre-agreed criteria to measure apparent enthusiasm of each participant—namely, the number of times that they answered questions, number of times that they asked questions, and number of times they participated with interest in discussions. It was measured in the first half of the session and second half of the session. Interest and enthusiasm of the participants was also noted, based on the level of interest and animation shown, as judged purely on the subjective impression of the research assistants. The level of interest shown in the first 5 min of the initial session conducted in each clinic was taken as the pre-status, while post status was the last 5 min of the second session.

### Identified and prioritized determinants

The determinants selected, when combined for all 26 clinics, yielded the following items as the most frequently mentioned across all settings. These were from the items that the participants offered, prior to added suggestions from the first author during advocacy process.

The following four determinants (Table 3) were the most commonly mentioned by the mothers’ groups initially.

**Table 3:** Determinants of LBW identified by the participants without mediation of the first author during facilitation process

1. Maternal nutrition 2. Work load/maternal rest	3. Poverty 4. Maternal infections

The items most commonly added following the first author’s facilitation are listed in Table 4.

**Table 4:** Determinants of LBW selected after the facilitation about determinants

1. Maternal happiness 2. Husband’s support 3. Care from other family members 4. Inequities (in household and community) 5. Attitudes of service providers	

Lack of knowledge among mothers and level of nutrition during the pregnancy were identified as immediate determinants for LBW, following the first authors’ explanation. Participants suggested lack of husbands’ support and inadequate family support as deeper level determinants. Abusive marital relationships were also identified as a hidden determinant and linked to lack of support from the husband. The mothers then chose the pregnant mother’s happiness (mental wellbeing) as a further important determinant for LBW. Inequities in the family or community setting and the negative attitudes among service providers were also offered as among the less evident determinants, in the latter part of the discussion.

Of items introduced by the first author, based on exiting studies, the most commonly selected are listed in Table 5.

**Table 5:** Determinants of LBW selected from The introduced determinants by the participants

1. Exposure to tobacco smoke 2. Indoor air pollution	3. Utilization of routine services 4. Care from the routine services

The items most commonly selected in the final list of 10 determinants are shown in Table 6.

**Table 6:** Determinants of LBW prioritized by the community

1. Maternal nutrition 2. Partner’s support 3. Exposure to tobacco smoke 4. Poverty 5. Indoor air pollution	6. Maternal happiness 7. Work load/maternal rest 8. Maternal infections 9. Care from other family members 10. Inequities (in household and community)

### Actions identified to address the prioritized determinants

Of around the 20 actions (range: 18 to 23) suggested as suitable to address the final list of determinants of LBW, there were some which also incorporated a method or tool to assess and some without. They are listed in Table 7.

**Table 7:** Actions identified to address the prioritized household level determinants of LBW

	Title	Description	Determinants addressed 1 = Maternal nutrition; 2 = Partner’s support; 3 = Maternal rest; 4 = Maternal happiness; 5 = Care by family; 6 = Indoor air pollution; 7 = Exposure to tobacco smoke; 8 = Poverty; 9 = Maternal infections; 10 = Inequities									
			1	2	3	4	5	6	7	8	9	10
1	Nutrition diary/calendar	Recorded the food consumed in each diet. Guided mothers to fulfill the daily nutrition requirements. Was adopted from the “Happy child diary” concept used in a child health promotion project [13].	X	X			X					X
2	Participation calendar	A tool to record and monitor the support received from family members by pregnant women. Commonly hung in the living room so that the others can monitor their participation in caring for the pregnant woman.	X	X	X	X	X					X
3	Happiness calendar	A tool to record and monitor the “happiness” of members of the household [13, 17].		X	X	X	X					X
4	Stimulation calendar	To record and monitor the activities to stimulate the fetus. Was adopted from “Happy Child diary”, a tool used to monitor the stimulation for children under 5 years [13].		X	X	X	X					
5	Expenditure diary	Recorded and monitored the expenditures of the family. This was divided into three categories. 1. “Sensible Expenditures”: those with positive consequences—nutritious food, education, improving housing and sanitations conditions etc. 2. “Not-so-sensible”: those with negative consequences—tobacco, alcohol, processed food, excessive sugar, fats etc. 3. Expenditures without negative consequences but not much in positive consequences and expenditures perceived as a “waste”—expensive clothing, unnecessary utensils, etc.	X					X	X	X		
6	*Vibhaga Pohora* (= “Brain Fertilizer”, to do well academically)	The homemade multiple micronutrient supplement was named such, based on the mothers’ hope of making the child clever. Includes heads of dried sprats (normally thrown away) roasted and mixed with an equal portion of dried *Moringa* leaves. The mixture ground to a powder, used as an additive when preparing flour based food or curries.	X	X			X					
7	Model menus	The menus comprised of locally available, low-cost food items with high nutrient value [20]. They were either prepared as “Set menus” (a meal with fixed food items), or based on “Food group charts”, from which participants can select food items for each meal.	X				X					
8	Food sharing	Is based on a common cultural practice among Sri Lankan communities. When a female becomes pregnant, relatives, friends and neighbors visit her with meals, comprised of food she prefers. This custom was enriched, so that the meal was of good quality and well balanced—based on “Model menus”.	X		X	X	X			X		X
9	Home gardening	A home garden, containing food identified in Model menus, especially dark green leafy vegetables was promoted. Partners, family members and neighbors helped in finding crops and maintaining the garden.	X	X			X			X		X
10	Reduction of processed food use	Aimed to shift the dietary habits of the households towards healthier options. Was also coupled with Model menus.	X							X		
11	“Smoke-free home”	Household level initiatives to stop smoking inside the house [18]. Stopping the idle gatherings of tobacco smoking visitors in the house; discouraging alcohol-centered ‘parties’; displaying posters on effects of tobacco smoke on the fetus, consequences of tobacco smoke to the smokers and non-smokers; verbal requests and discussions with the smokers to motivate them to quit were the actions implemented				X	X	X	X		X	
12	Reduction of tobacco use	Was coupled with “Smoke-free-homes”. Apart from the actions mentioned, reducing availability of cigarettes by motivating the local vendors to stop selling them; improving awareness on the industrial influence by poster campaigns and informal discussions; reducing smoking in public places using poster campaigns and informal discussions were the other actions implemented.	X			X		X	X	X		
13	Reduction of alcohol use	Was coupled with reduction of tobacco use and used the same strategies. Additionally, myths on alcohol related behaviors and undue privileges for users were also addressed [19].	X			X		X	X	X		
14	Remodeling kitchens	Cleaning chimneys, establishing vents and windows to improve air flow, replacing open fires with bio-mass efficient cookers were implemented. Partners played a major role and the expenses were covered by the money saved from cutting down unnecessary and unhealthy expenditures as explained under the “Expenditure diary” (#5, above).		X				X				
15	*Siriyavantha Nivasa* = A pleasant house	Aim was to generate a clean and safe environment. Improving illumination and ventilation, re-organizing furniture to improve space and avoid accidents, keeping the floor clean of clutter and free of dust, keeping the garden clean and free of mosquito breeding sites, making the house a pleasant place by hanging family portraits and pictures on nature and discouraging loud music being played on record players were the common actions implemented.			X	X	X	X	X		X	
16	Pregnant mother’s room	Was adopted from “Baby room” concept [13]. Participant, partner and the family and friends engaged in preparing the room for the newborn. The purpose was to create a room or space with adequate illumination and ventilation, free of tobacco smoke, clean, and decorated to provide stimulations for the new born.		X	X	X	X		X			
17	Listening to lullabies	In addition to providing stimulation for fetal development, listening to lullabies and other soft, soothing music, was aimed at improving maternal mental wellbeing and bonding with the newborn. Mothers were encouraged to sing it by themselves or together with partners, older children and other family members. Lullabies were collectively sung in collective play houses as well.		X	X	X	X					
18	Collective play houses	Mothers living nearby gathered every evening in a play house to engage in interactive play with the children [13]. The play house was rotated among the group members, thus, the mother owning the house got the leadership to organize the sessions. As mothers’ capacities differ, all the children in the community got equal opportunities for stimulation. Presence of neighbors in the households in the evenings prevented smoking and drinking “parties” and post-drunkard violence [15]. Partners and other family members got involved in building the play houses and engaged in its activities. As older children were engaged every evening in the play house, pregnant women were relieved of taking care of them and had an opportunity to rest.		X	X	X	X					
19	Collective feeding	Aim was to provide an adequate, good quality meal for all children at least once a day. Commonly coupled with collective play houses. Mothers prepared the meal as a group, sharing the resources, ensuring its quality, diversity and adequacy. Children fed as a group were found to eat better than alone, playing and stimulating each other [13].			X	X	X			X		X
20	Interactions with nature	Spending time in the home garden, looking at birds and butterflies, enjoying the night sky, nature expeditions to local settings such as lakes were the actions promoted. Participation of other children, partners and family members in those interactions was also encouraged. Aim was to facilitate bonding between the family members and improve family wellbeing and provide stimulation for older children.		**X**	**X**	**X**	X					

## Discussion

The key finding from this study is that rural mothers engaged successfully in a process to identify determinants of LBW, work out suitable interventions to address these, and devise appropriate indicators to assess changes in them. The main reason for success in this process was probably the interactive approach used. It relied on giving leadership and control to the mothers who participated, while supplementing their discussions with technical knowledge derived from existing scientific studies. Mothers readily understood and accepted the need to examine underlying factors, in order to create desired changes. They were quickly able to suggest immediate or proximal determinants of LBW. And they used their collective judgment to accept or reject ideas derived from existing scientific studies. They were quick to analyze and incorporate new ideas that were suggested to them, and take on board those that they considered relevant and useful.

Community members identified determinants of LBW applicable to their own community—some of which corresponded to those found in the scientific literature. These included the nutritional state of the mother, the type and amount of work and rest during pregnancy, care received by the pregnant woman, and her emotional status identified—which correspond with other studies (Kramer, 1987). Most determinants suggested to them from the existing literature were accepted by community members as relevant to their community also. For example, community members added environmental tobacco exposure and indoor air pollution as determinants, which were suggested to them based on previous studies (Ohlsson and Shah, 2008). Community members also identified as determinants several factors established from Sri Lankan settings (Abeysena, 1995; Abeysena, 2002; Abeysena et al., 2002; Abeygunawardena, 2010; Jazeelul Illahi, 2007; Samarasinghe, 2006; Ruwanpathirana, 2011; UNICEF, 2006b; Wijayawardena et al., 2010). Determinants newly suggested by members of the community included care from the routine health services.

Mothers then went on to determine a range of feasible, integrated, low-cost actions to address multiple household level determinants of LBW as well as suggest practical indicators to measure changes in these. Here too, a significant ingredient of success was the combination of their real-life wisdom and the first author’s technical contribution.

One of the strengths of this study is that assessments by the researchers are validated by ongoing monitoring and assessment of progress by the community members. The records maintained by mothers’ groups are not included in this report but provided the researchers invaluable corroboration of their conclusions. That the process was led by the community itself—in keeping with how a health promotion intervention should be implemented—allow participants to move on smoothly to actual intervention. The readiness with which new information was assimilated by the community is also a benefit that the shared and interactive methodology yielded. The increase in husbands’ participation may have been due to community-wide effects, as more than one family from each small locality was addressed.

A weakness in the assessment of issues such as apparent interest and enthusiasm of mothers is that they rely on highly subjective measurements. The attempt to reduce the impact of errors due to subjectivity through role plays and giving research assistants the opportunity to observe each other’s ratings may not have adequately removed errors due to subjectivity—even though there was no inducement for research assistants to engage in systematic bias in one direction or another. This process indicator was used, despite its reliability being open to question, as it is a measure that the community found meaningful. The usefulness of such measures requires that they are retained, for they serve also as a tool that strengthens the community’s ability to assess their progress. In the hands of the community, such assessments become increasingly reliable as they continue to use them and question their own results critically.

A drawback of using subjective assessments such as this is that they make it difficult for others to apply the same methods in other settings. But this should not be considered a real weakness. Replication in these kinds of interventions should be of the broad process, rather than of the specifics of measurement. Measurement is itself a tool in mobilizing communities.

The groups did move on to applying to their communities the conclusions they made regarding determinants of LBW. The main conclusion from this study is that mothers with little formal education and no training in health interventions or research did succeed in deciding on determinants and designing suitable interventions and measures of change, with the appropriate technical input. The lesson from this is that theoretical and technical inputs are rapidly integrated and used when they are delivered in the right context—namely, as part of the community’s own effort to learn and intervene.

The findings suggest that this approach can be a useful supplement to the package of ante-natal interventions currently carried out by the field health services of the Ministry of Health. Including the approach used in this study can be recommended for inclusion as a part of routine ante-natal clinic activity, as there has been a major beneficial response. To make it feasible, the time taken for each session can be shortened while the intervention can be spread over a larger number of sessions.

Present interventions through field health staff do not provide for an active role for mothers and communities in formulating strategies to reduce low birth weight. They rely more on providing information and giving feedback through monitoring of pregnant mothers and children.

## Conclusions

The relative ease with which mothers’ groups took up the idea of analyzing underlying causes or determinants and the ease with which they moved to working out ways of addressing these shows that lay people are quite well equipped to deal with such seemingly sophisticated tasks. This capacity can usefully be engaged in addressing LBW in communities similar to those studied.

A cautious recommendation can be made that field health staff are further trained in the interactive “health promotional” approaches used here. We need to study whether further training of a selected group of current health sector staff, in the approaches used in this study, will lead to their applying the relevant principles and skills in their routine work. Another area that merits study is of the spinoff benefits, to other aspects of family and community wellbeing, through mothers coming together to address LBW.

## Abbreviations

ANC: Antenatal clinic
LBW: Low birth weight
MOH: Medical Officer of Health
POA: Period of amenorrhea
